# Supplementary material for: Power output from 12 brands of contemporary LED light-curing units measured using 2 brands of radiometers
Source: PLoS One. 2022 Jul 8;17(7):e0267359. doi: 10.1371/journal.pone.0267359 (PMC9269870; doi:10.1371/journal.pone.0267359)
Supplement: Attachment — Submitted filename: Reviewers comments .doc [file pone.0267359.s002.doc]

1.

In citation,

29. Worthington HV, Khangura S, Seal K, Mierzwinski-Urban M, Veitz-Keenan A, Sahrmann P, et al. Direct composite resin fillings versus amalgam fillings for permanent posterior teeth. Cochrane Database Syst Rev. 2021;8:CD005620. Epub 20210813. doi: 10.1002/14651858.CD005620.pub3. PubMed PMID: 34387873; PubMed Central PMCID: PMCPMC8407050.

There’s your sentences:

M E T H O D S

Criteria for considering studies for this review

Types of studies

For the outcome of efficacy, we included randomised controlled trials (RCTs) comparing dental composite resin with dental amalgam restorations in permanent posterior teeth (dating back to 1946). We excluded studies that had less than a three-year followup period.

*Direct composite resin fillings versus amalgam fillings for permanent posterior teeth . This a*rticle written in 2021,but the cited literature’s date is too old, which I think is inappropriate, and I suggest you cite another references.

2.

As the LCU usage time increasing, the attenuation of the radiant output should also be taken into account.

3.

The effect of voltage on light intensity should be considered. For example, a 100% battery will produce the highest voltage intensity. A battery with a 10% charge will produce a smaller voltage.

In clinical, it is impossible for us to keep the LCU at 100% power all the time.

4.

The following sentence might be a sick sentence:  For LCU units #1, the following lights showed percentage differences beyond 10%: Dental Spark, SDI Radii Plus and Woodpecker LED.B. For LCU units #2, these lights showed percentage differences beyond 10%: Dental Spark, SDI Radii Plus, Woodpecker LED. B and Woodpecker LED.D.

5.

Did you consider the effect of temperature on the test during the experiment?

6.

The photoinitiator system is composed of photoinitiator and co-initiator, which plays a decisive role in the photocuring rate of the material. The most commonly used photoinitiator is camphoroquinone (CQ, camphoroquinone), the addition amount is usually 0.05%-1% (mass fraction). Camphorquinone absorbs blue light in the range of 400-500 nm, with a maximum absorption peak at 470 nm.

The entire article focuses on the radiated power of LCUs. I think the peaks of each spectrum are the ones to focus on.

More attention should be paid to clinical practice. What we want is the curing speed and depth, not the rigid value of radiation power.
