# Supplementary material for: Power output from 12 brands of contemporary LED light-curing units measured using 2 brands of radiometers
Source: PLoS One. 2022 Jul 8;17(7):e0267359. doi: 10.1371/journal.pone.0267359 (PMC9269870; doi:10.1371/journal.pone.0267359)
Supplement: Attachment — Submitted filename: Response to Reviewers .docx [file pone.0267359.s003.docx]

February 28, 2022

**PLOSOne**

**Dr. Zhiyuan Zhu**
Southwest University
CHINA

Dear Dr. Zhu:

Revisions to:

PONE-D-21-40118R1
Light Output from 12 Brands of Contemporary LED Light-Curing Units Measured Using 2 Brands of Radiometers

Enclosed is our revised manuscript that we would like to resubmit to PLOS One. The purpose of the article is to alert your readers to four important points:

1. Using a malfunctioning or an inadequate dental curing light has an economic impact estimated to cost billions of dollars annually.
2. Dental curing lights should be tested regularly.
3. The Bluephase Meter II can accurately measure the power from most dental curing lights and should be used by dentists
4. If the researcher cannot afford and cannot access optics laboratory equipment, the Mini Gig radiometer should be used. This will provide the power, spectral radiant power and the emission spectrum.

All the authors have reviewed, discussed, and agreed to their individual contributions.

**The Acknowledgments Section of our manuscript should state:**

‘The authors received no specific funding for this work. Dr. Cristiane Maucoski is a visiting graduate student from Brazil. She received a travel grant provided by a MITACS award IT26826 for C. Maucoski and an internal research fund grant from the Faculty of Dentistry, Dalhousie University. The study was also supported by grant provided by Coordination for the Improvement of Higher Education Personnel (CAPES grant # 88881.622852/2021-01).
The funders had no role in study design, data collection and analysis, decision to publish, or preparation of the manuscript.’

We have uploaded a clean and a tracked version, but to answer your reviewer’s specific comments:

1. We apologize for any confusion. Dr. Cristiane Maucoski is visiting from Brazil. She received a Globalink grant of $6,000 from MITACS to come to Canada. The money was not specific to the research presented in the manuscript, but without the MITACS Globalink support she could not have come to Canada. As such, we believe that the following ‘Funding Information’ and ‘Financial Disclosure’ statements are now correct. ‘The authors received no specific funding for this work. Dr. Cristiane Maucoski is a visiting graduate student from Brazil. She received a travel grant provided by a MITACS award IT26826 for C. Maucoski and an internal research fund grant from the Faculty of Dentistry, Dalhousie University. The study was also supported by grant provided by Coordination for the Improvement of Higher Education Personnel (CAPES grant # 88881.622852/2021-01).
   The funders had no role in study design, data collection and analysis, decision to publish, or preparation of the manuscript.’
2. To address the concerns that the 2021 Cochrane review was too old, we have added additional references and reworded the paragraph. However, the meaning is still the same, RBCs do not last as long as they should. Why?

This may explain why a Cochrane review published in 2021 reported that direct resin-based composite (RBC) restorations placed in posterior teeth have almost double the failure rate of amalgam restorations[31]. Several previous publications have reported higher failure rates for RBCs [18, 32] and that the median longevity of posterior RBCs is less than 7 years [33, 34].

3. To address the concern that as the LCU usage time increasing, the attenuation of the radiant output should also be taken into account. And The effect of voltage on light intensity should be considered. For example, a 100% battery will produce the highest voltage intensity. A battery with a 10% charge will produce a smaller voltage.
   We addressed this when we wrote ‘The LCUs were recharged after every 5 exposures to ensure that their batteries were always adequately charged.’
4. To address the concern that For LCU units #1, the following lights showed percentage differences beyond 10%: Dental Spark, SDI Radii Plus and Woodpecker LED.B. For LCU units #2, these lights showed percentage differences beyond 10%: Dental Spark, SDI Radii Plus, Woodpecker LED. B and Woodpecker LED.D, we revised the sentence to read: Examples #1 and 2 of the Dental Spark, SDI Radii Plus and Woodpecker LED.B both showed percentage differences in the power output that were beyond 10%. Only example #2 of the Woodpecker LED.D had percentage differences in the power output that were beyond 10%. We reworded the section to read:

**3.3 Comparison between LCUs**

Figures 7 and 8 illustrate the percentage differences of each radiometer compared to the ‘GS’ measurement for each LCU. Of note, both meters reported power values that were above and below the ‘GS’ value. Examples #1 and 2 of the Dental Spark, SDI Radii Plus and Woodpecker LED.B both showed differences in the power output that were more than 10% different from the ‘GS’. Example #2 of the Dental Spark, SDI Radii Plus, Woodpecker LED.B and the Woodpecker LED.D had percentage differences in the power output that were greater than 10% different from the ‘GS’. Apart from the SDI Radii Plus, we considered these to be 'budget lights’. Figures 7 and 8 show that the highest percentage difference was for the Pinkwave when using the Bluephase Meter II, and these values were obviously incorrect. For the Mini Gig meter #1, the greatest difference from the 'GS’ result was 9%. For the Mini Gig meter #2, the greatest difference from the 'GS' was 15%. Both of these values were from the budget light Dental Spark LCU (c), whose standard deviations in the ‘GS’ power values were also large (±25 and 30 mW).

5. To address the concern, Did you consider the effect of temperature on the test during the experiment? We added:

The LCUs and meters were used in random order and at room temperature (20°C±1) to mimic the condition in a dental office.

6. To address the concern, More attention should be paid to clinical practice. What we want is the curing speed and depth, not the rigid value of radiation power. We believe that this reviewer has missed the point. Dentists and researchers need a reliable and accurate instrument to measure the light output from their light. This article tells the dentist and the researcher what to buy. The dentist ‘should be confident purchasing the Bluephase Meter II because it could accurately report the power from 11 out of the 12 brands of LCU tested.’ The researcher should purchase the Mini Gig. We have significantly edited the manuscript and hoe that this meets the reviewer’s concerns. Following our recommendations will enable dentists to make better treatment decisions that are based on the power (Watts) and amount of energy (Joules) that can be delivered to the RBC from the LCU.
7. We thank the reviewer for pointing out that we had not described what RBC meant before using the term. This error has been corrected.
8. We thank the reviewer for pointing out that we had not described Table 2 in the text. This has been corrected.
9. If our manuscript is accepted for publication, we shall upload our laboratory protocols to [protocols.io](http://protocols.io) to enhance the reproducibility of our results.  In the meantime, we have uploaded them as a compressed folder as requested.

Sincerely yours,

R.B. Price, BDS, DDS, MS, FRCD(C), PhD

Professor of Prosthodontics
