# Supplementary material for: Power output from 12 brands of contemporary LED light-curing units measured using 2 brands of radiometers
Source: PLoS One. 2022 Jul 8;17(7):e0267359. doi: 10.1371/journal.pone.0267359 (PMC9269870; doi:10.1371/journal.pone.0267359)
Supplement: Attachment — Submitted filename: Reviewers comments2.doc [file pone.0267359.s004.doc]

1.

There’s your sentences:

Of note, in Figure 3, the Pinkwave LCU would remain on for 20-s and the Radii Plus for 60-s, but when tested, they were manually turned off after 10-s.

Have you ever pressed a button manually？If you press a button with your finger, how do you make sure the timing of each button press is very accurate?

2.

There’s your sentences:

Figure 4 shows that 7 (a to g) of the LCUs: (SmartLite Pro, DeepCure S, Dental Spark, Denjoy, Woodpecker LED B and D, and Radii Plus) were single emission peak LCUs. The remaining 5 LCUs (h to l) were broader spectrum multi-emission peak LCUs. The Pinkwave LCU (l) emitted 4 distinct wavelength peaks, between the range of 350 – 870 nm (λ1 = 411 nm, λ2 = 472 nm, λ3 = 633 nm, and λ4 = 857 nm) and was notably different from the other LCUs.

Do you have the spectrogram similar to this picture below?If the answer is yes, please add into the article.
