# Supplementary material for: Power output from 12 brands of contemporary LED light-curing units measured using 2 brands of radiometers
Source: PLoS One. 2022 Jul 8;17(7):e0267359. doi: 10.1371/journal.pone.0267359 (PMC9269870; doi:10.1371/journal.pone.0267359)
Supplement: Attachment — Submitted filename: Response to Reviewers R2 .docx [file pone.0267359.s005.docx]

April 4, 2022

**PLOSOne**

**Dr. Zhiyuan Zhu**
Southwest University
CHINA

Dear Dr. Zhu:

Revisions to:

PONE-D-21-40118R2
Light Output from 12 Brands of Contemporary LED Light-Curing Units Measured Using 2 Brands of Radiometers

Enclosed is our revised manuscript that we would like to resubmit to PLOS One.

Comment #1:

Have you ever pressed a button manually？If you press a button with your finger, how do you make sure the timing of each button press is very accurate?

Comment #2:

Do you have the spectrogram similar to this picture below? If the answer is yes, please add into the article.

Answers:

1. We have addressed the concern about the 10-s. The exact timing was not relevant to the study, but we have addressed this concern in the text.
2. As requested, we have added a colour spectrum to Figure 4 and added it as a striking image.

The authors received no specific funding for this work, but the authors wish to thank the manufacturers for the loan of their Bluephase Meter II and the Mini Gig radiometers used in this study.

Dr. Cristiane Maucoski is a visiting graduate student from Brazil. She received a travel grant provided by a MITACS award IT26826 and an internal research fund grant from the Faculty of Dentistry, Dalhousie University. Her visit to Canada was also supported by grant provided by Coordination for the Improvement of Higher Education Personnel (CAPES grant # 88881.622852/2021-01). Neither the manufacturers of the radiometers nor the funders of Dr. Maucoski’s visit to Canada had any role in the study design, data collection and analysis, decision to publish, or preparation of the manuscript.

Sincerely yours,

R.B. Price, BDS, DDS, MS, FRCD(C), PhD

Professor of Prosthodontics
